# The Cross‐Linguistic Coordination of Overt Attention and Speech Production as Evidence for a Language of Vision

**DOI:** 10.1111/cogs.70185

**Published:** 2026-02-23

**Authors:** Moreno I. Coco, Eunice G. Fernandes, Manabu Arai, Frank Keller

**Affiliations:** ^1^ Department of Psychology Sapienza University of Rome; ^2^ I.R.C.S.S Fondazione Santa Lucia; ^3^ School of Psychology University of Minho; ^4^ Faculty of Economics Seijo University; ^5^ School of Informatics University of Edinburgh

**Keywords:** Cross‐modal coordination, Scene description, Cross‐linguistic differences, Semantic and syntactic similarity, Eye‐tracking, Language of vision, Scene grammar

## Abstract

A central question in cognition is how representations are integrated across different modalities, such as language and vision. One prominent hypothesis posits the existence of an abstract, prelinguistic “language of vision” as a representational system that organizes meaning compositionally, enabling cross‐modal integration. This hypothesis predicts that the language of vision operates universally, independent of linguistic surface features such as word order. We conducted eye‐tracking experiments where participants described visual scenes in English, Portuguese, and Japanese. By analyzing spoken descriptions alongside eye‐movement sequences divided into planning and articulation phases, we demonstrate that semantic similarity between sentences strongly predicts the similarity of associated scan patterns in all three languages, even across scenes and between sentences in different languages. In contrast, the effect of syntactic constraints was secondary and transient: it was restricted to within‐language and within‐scene comparisons, and temporally confined to the early planning phase of the utterance. Our findings support an interactive account of cross‐modal coordination in which a universal language of vision provides stable semantic scaffolding, while syntax serves as a local constraint, primarily active during message linearization.

## 1 Introduction

A longstanding debate in cognitive science concerns the existence and nature of a language of thought (LoT). First introduced by Fodor (1975), the LoT is hypothesized to have a compositional structure consisting of simple concepts that combine systematically to form more complex mental representations. These representations underpin cognitive processes across various modalities, including language, visual perception, and reasoning. Open questions regarding the LoT include whether its representations are symbolic or nonsymbolic, whether its combination rules are logical or probabilistic, and how patterns of human responses, including neural activity, reflect aspects of the LoT (Quilty‐Dunn, Porot & Mandelbaum, 2023; Kazanina & Poeppel, 2023; Doerig, Sommers, Seeliger, Richards, Ismael, Lindsay, Kording, Konkle, van Gerven, Kriegeskorte & Kietzmann, 2023). Researchers have often explored the LoT through the lens of human language, particularly at the intersection of syntax and semantics (Jackendoff, 1992). However, language is a relatively recent product of human evolution (Corballis, 2017). Perception, a much older cognitive component, could instead be responsible for organizing causal relationships between events (e.g., agent–patient interactions) into structured chunks of meaning (Hespos & Spelke, 2004; Wilson, Zuberbühler & Bickel, 2022). In this view, attentional mechanisms directly construct representations that other cognitive processes can access, driven by an underlying “language of vision” (LoV), assumed to be an instance of the LoT (Cavanagh, 2021).

Drawing on this framework, we operationally define the LoV as the visual information sequentially apprehended via oculo‐motor responses while speech is planned and articulated. This definition reflects the idea that attention structures perceptual information into coherent messages shared with other cognitive processes, such as memory, language, and reasoning (see Gregory (1974) for the initial seeds of this idea). This aligns with findings demonstrating the crucial role of attention‐motor coordination in motor control (e.g., object fixation before action Land, Mennie & Rusted 1999; Land 2012; Hayhoe, McKinney, Chajka & Pelz 2012; Foulsham, Walker & Kingstone 2011), facilitating successful learning (Pagnotta, Laland & Coco, 2020), and joint action between individuals (Clark & Krych, 2004; Coco, Dale & Keller, 2018; Galati, Dale, Alviar & Coco, 2026; Hessels, Teunisse, Niehorster, Nyström, Benjamins, et al. 2023; Louwerse, Dale, Bard & Jeuniaux, 2012). Furthermore, the influence of visual information on language is well‐established, with evidence suggesting that spatial relationships among objects or figure‐ground separation shape language (Talmy, 1975; Landau & Jackendoff, 1993) and operate within universal constraints shared cross‐culturally (Coventry, Gudde, Diessel, Collier, Guijarro‐Fuentes, Vulchanova, Vulchanov, Todisco, Reile, Breunesse & others, 2023). Moreover, visual information exhibits statistical regularity and is predictable (Kaiser, Quek, Cichy & Peelen, 2019)—fundamental characteristics that enable tasks such as categorization (Bar, 2004; Greene & Oliva, 2009; Thorpe, Fize & Marlot, 1996), memory recognition (Bainbridge, Hall & Baker, 2019; Evans & Baddeley, 2018), or visual search (Draschkow & Võ, 2017; Brockmole & Henderson, 2006; Torralba, Oliva, Castelhano & Henderson, 2006). The regularity of visual information has led researchers to postulate an underlying “scene grammar” (Võ, 2021; Võ, Boettcher & Draschkow, 2019), an idea closely related to the LoV hypothesis. Support for this concept comes from evidence demonstrating that violations of structural or conceptual regularities in scenes affect human visual processing, as evidenced by both behavioral (Coco & Duran, 2016; Davenport & Potter, 2004; Wolfe, Alvarez, Rosenholtz, Kuzmova & Sherman, 2011) and neurophysiological responses (Coco, Araujo & Petersson, 2017; Coco, Nuthmann & Dimigen, 2020; Lauer, Cornelissen, Draschkow, Willenbockel & Võ, 2018; Mudrik, Lamy & Deouell, 2010; Mudrik, Shalgi, Lamy & Deouell, 2014; Võ & Wolfe, 2013).

Postulating an LoV entails adopting a variant of the LoT hypothesis, which posits that mental representations possess a compositional structure and combine in systematic, rule‐governed ways. Such representations enable the cognitive system to coordinate across distinct sensory and cognitive modalities. While their nature may be symbolic or subsymbolic, LoT representations are fundamentally semantic, encoding the meaning of information rather than its surface form (e.g., the specific words or the syntactic structure of a sentence). Consequently, the key prediction of the LoV hypothesis is that the coordination between modalities is universal across languages and is primarily driven by semantic, rather than syntactic, representations. In practice, the grammatical driver of meaning building is the compositional oculo‐motor routine that the attentional system operates to feed the concurrent linguistic process. The influence of an LoV should, therefore, manifest itself throughout the entire speech process, be independent of its surface realization, and generalize from the specific details of the visual context that is linguistically encoded. Thus, a crucial expectation from this hypothesis is that coordination between speech generation and visual attention during tasks such as image description is primarily driven by the semantic content of utterances rather than their syntactic form. This implies that task performance should be consistent across languages with significantly different syntactic properties. In essence, cross‐modal coordination between vision and language is primarily enabled by a global semantic scaffolding provided by an LoV.

Individual languages exhibit substantial variability in their syntactic properties, such as word order, which could influence the coordination between visual attention and speech processes across different languages. Evidence supporting this view comes from research using the visual world paradigm (VWP; Cooper 1974; Tanenhaus, Spivey‐Knowlton, Eberhard & Sedivy 1995), which examines the incremental mechanisms of phrase building during spoken‐language comprehension, as inferred from anticipatory eye movements toward targets in a visual context. Crucially, syntactic information including grammatical function (Altmann & Kamide, 1999; Knoeferle & Crocker, 2006; Coco, Keller & Malcolm, 2016), subcategorization (Arai & Keller, 2013), case marking (Kamide, Altmann & Haywood, 2003; Kamide, Scheepers & Altmann, 2003), and tense (Altmann & Kamide, 2007) is used to guide this process. A similar relationship is observed in speech generation, where visual referents are fixated just before they are mentioned (Griffin & Bock, 2000; Bock, Irwin, Davidson & Levelt, 2003; Kuchinsky, Bock & Irwin, 2011) and the associated timings modulated by the perceptual characteristics of scenes, but also by the syntactic properties of the sentences being produced (Coco & Keller, 2015), including grammatical function (Griffin & Bock, 2000) or word order (Brown‐Schmidt & Tanenhaus, 2006). Based on these results, we may expect that the syntax of an utterance is a critical determinant of cross‐modal coordination, and differences in attentional behavior should emerge between speakers of different languages performing the same scene description task. Indeed, VWP comparisons of the eye‐movement patterns of English speakers with those of Japanese speakers (Kamide et al., 2003), German speakers (Kamide et al., 2003), and Spanish speakers (Brown‐Schmidt & Konopka, 2008) provide evidence for this expectation. In essence, a strict “syntactic hypothesis” would posit that the cross‐modal coordination between vision and language primarily reflects the surface form of inputs and outputs. Crucially, this influence extends beyond linear word order to encompass hierarchical structure, for example, as formalized by dependency syntax. Under this view, the grammatical complexity and structural relations—such as those between heads and their dependents—should predict how overt attention is allocated to a scene, above and beyond the semantics of the message being conveyed.

Beyond these two extremes, where coordination could be governed by either purely semantic or purely syntactic constraints, interactionist accounts focus on the way they interface, assuming a constraint‐based dynamical interaction between semantic inputs and syntactic forms (e.g., Myachykov, Posner & Tomlin (2007); see also Jackendoff (2025) for a recent theoretical review). Within this framework, the perceptual organization of visual information into an event structure is influenced by the syntactic organization of relations (thematic, spatial, or action‐related) (Gleitman, January, Nappa & Trueswell, 2007; Papafragou, Hulbert & Trueswell, 2008; Myachykov, Thompson, Scheepers & Garrod, 2011; Hafri, Gleitman, Landau & Trueswell, 2022; Ünal, Wilson, Trueswell & Papafragou, 2024). Crucially, the predictions of such interface models depend on the time course of speech generation. If an LoV encodes a universal event structure (Mani & Johnson‐Laird, 1982), overt attention should consistently prioritize semantic roles, independent of the phase of the language production process. However, if syntax acts as a local constraint on this process, then its influence should be transient—necessary only when the message is linearized into a sentence using the grammar of a specific language. Under the interactive account, we would, therefore, predict a distinction between the planning phase in language production (the conversion of nonlinear meaning into a linear sequence; Lashley & others 1951; Dell, Burger & Svec 1997) and the subsequent articulation phase (the transition from conceptualization to articulation; Levelt, Roelofs & Meyer, 1999). The generalized influence of semantic representations aligns with the event‐perception literature, which demonstrates the rapid, automatic extraction of relational event structure from visual input (Zacks & Tversky 2001; see Hafri & Trueswell 2025 for a recent review).

The present study evaluates three competing theoretical positions regarding the cross‐modal coordination of vision and language: the LoV hypothesis, a strictly syntactic hypothesis, and the hybrid interactionist model. We build on the results of Coco and Keller (2012), who demonstrated a strong relationship between linguistic production and visual attention. In their study, English‐speaking participants were eye‐tracked while viewing photorealistic scenes and producing verbal descriptions after being cued with an object label. The hypothesis was that speakers who produce similar verbal descriptions also show similar patterns of overt attention. This was tested by comparing the scan patterns (sequences of fixated objects) across participants and scenes using the longest common subsequence (LCS) measure. A regression analysis then showed that sentence similarity predicts scan pattern similarity, both within and across scenes, suggesting strong coordination between visual attention and speech generation. However, this prior work was limited to English and utilized surface‐level linguistic measures, failing to distinguish between semantic factors and hierarchical syntactic constraints. Consequently, it could not address whether this coordination is cross‐linguistically consistent or dependent on specific grammatical structures.

**Fig. 1:**
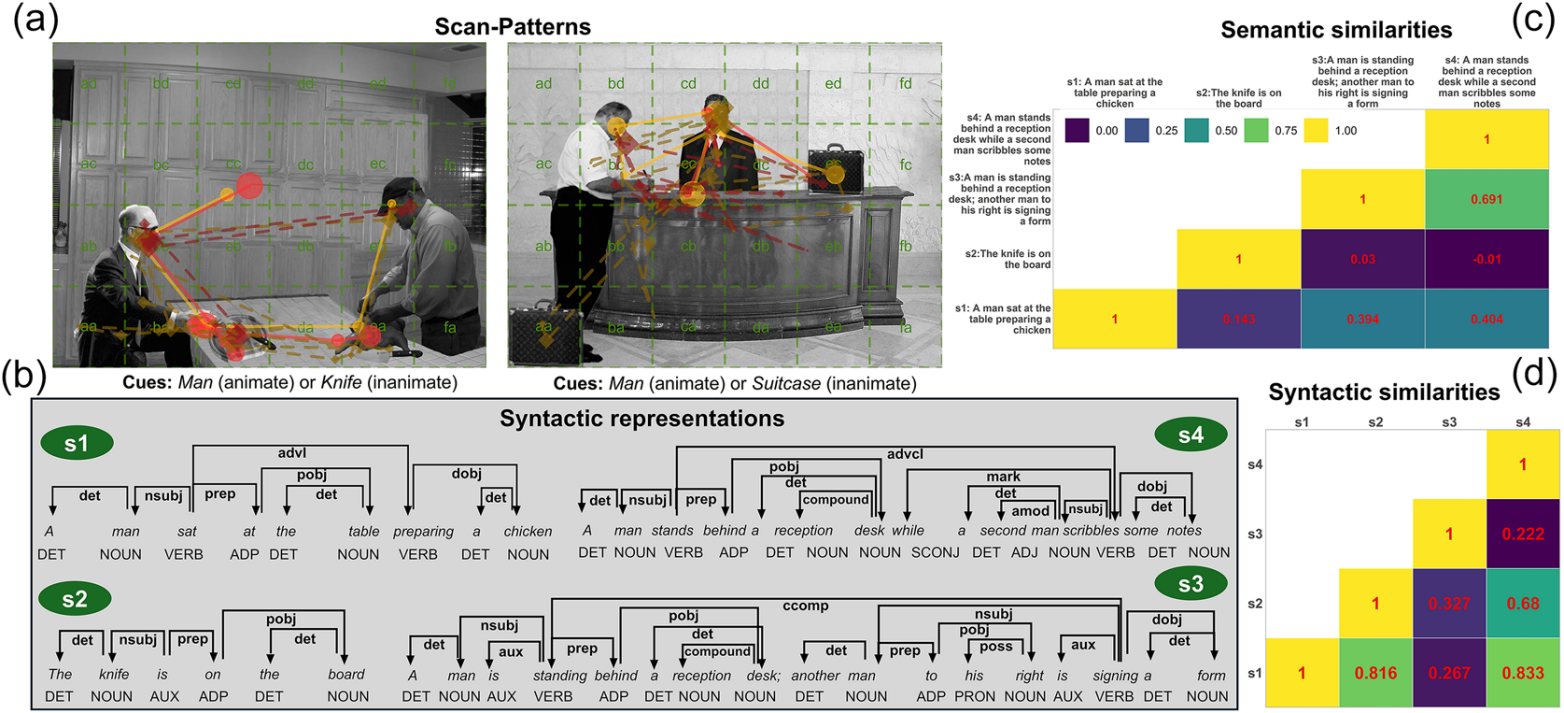
Example of eye‐movement and linguistic data of four trials for English speakers in two different scene contexts, and associated similarity metrics. (a) Examples of scenes (in black and white) with overlaid cell grids to map fixation coordinates into categorical sequences of fixated locations. In each scene, we plot the scan patterns of the speakers using color (red and yellow), point and line types to distinguish the underlying phases of speech production (circle‐solid line, before speaking; diamond‐dashed line, during speaking). The lines mark the order of fixated locations. (b) Dependency trees of the four sentences produced in the visualized trials. (c) Pairwise semantic similarity across the four sentences, computed using the Universal Sentence Encoder (Cer, Yang, Kong, Hua, Limtiaco, John, Constant, Guajardo‐Cespedes, Yuan, Tar, Sung, Strope, & Kurzweil, 2018). (d) Pairwise syntactic similarity across the four dependency trees using tree kernels computed over dependency trees (Moschitti, Pighin & Basili, 2008).

To address these limitations, the current study compares scene descriptions across two distinct high‐dimensional spaces: semantic similarity (via semantic embeddings) and syntactic similarity (via Dependency Tree Kernels), across three languages with varying word orders: English (SVO), Portuguese (SVO, modifier‐final), and Japanese (SOV). If the LoV (or a universal LoT) is the primary representational system guiding attention, we expect scan patterns to be consistently associated with semantically similar descriptions across all languages, regardless of syntactic variance, throughout the entire speech generation process. Conversely, if syntactic constraints are the primary drivers, then cross‐modal coordination should track hierarchical syntactic dependencies and diverge based on language‐specific constraints (e.g., English verb‐medial vs. Japanese verb‐final structures). For the syntactic hypothesis, it is challenging to predict which phase of speech production (planning or articulation) is most impacted by scan pattern similarity—effects in the literature are observed in both phases, albeit for different tasks (i.e., comprehension and production). Finally, an interactionist account of the language–vision interface would be supported if we observe a dynamic interplay in which global semantic guidance is modulated by syntactic constraints, particularly during the early planning phase of message formulation. Fig. 1 illustrates the logic of our experiment and provides examples for scenes, scan patterns, and sentences.

## 2 Method

In this eye‐tracking study, participants were asked to verbally describe visual objects embedded in photorealistic pictures of indoor scenes. Before they saw each visual scene, the object to be described was prompted with a cue word, which denoted either an animate (e.g., man) or an inanimate (e.g., knife) referent. The advantage of this generation constraint, compared to free production, is that it encourages participants to focus on describing relationships between the visual elements present in the scene, explicitly anchoring overt attention to the linguistic descriptions (i.e., participants do not refer to information not visually present or events not contingent on available stimuli). We collected participants' eye movements from the moment the scene appeared on the screen, capturing the planning process, until they articulated an utterance and indicated that their description of the cued object was complete by pressing the space bar to move to the next trial.

### 2.1 Design

The experimental design crossed scene Clutter (Minimal vs. Cluttered) and the Animacy of the visual target (Animate vs. Inanimate). Additionally, the experimental materials were designed such that each cue word could ambiguously refer to two visual objects in the scene. In the current study, however, we do not analyze these specific experimental manipulations, as the influence of factors such as clutter, animacy, and ambiguity is tangential to our primary theoretical objectives and has been examined elsewhere (Coco & Keller, 2015).

### 2.2 Participants

Seventy‐four participants (24 speakers of British English, 13 Female, age = 23.08±3.99; 28 speakers of European Portuguese, 20 Female, age = 20.83±3.39; 20 speakers of Japanese, 17 Female, age = 23.65±4.30) took part in the study. The English speakers were all students of the University of Edinburgh and paid 5 pounds for their participation. The data for this sample have already been published in Coco & Keller (2012). The Portuguese and Japanese speakers were students at the University of Lisbon and volunteered as unpaid participants. The Japanese speakers had resided in Portugal for an average of 5.87±4.70 months prior to data collection, a relatively short duration that makes attrition of their native language unlikely.

Power analysis to estimate the sample size was computed using the approach by Green & MacLeod (2016), designed to handle linear‐mixed effects models. Specifically, we estimated the observed power from the data published in Coco & Keller (2012) by fitting a linear‐mixed model predicting scan pattern similarity as a function of sentence similarity, as in the original paper. We aggregated across participants (introduced as random effects) to make simulations computationally tractable on a standard PC (i.e., reducing from 123,256 data points to 576). Even after this reduction, we obtained an estimated power of 100% (CI = 99.63%–100%) with an effect size of 0.2, indicating that scan pattern similarity is predicted by semantic similarity. Moreover, simulations to explore the trade‐off between sample size and power indicate that we need 21 participants to reach a power of 88% (CI = 86.68%–90.69%) to detect a fixed effect of semantic similarity with an effect size of 0.2.

The study adhered to the 1964 Declaration of Helsinki. The ethics committees of the University of Edinburgh and the University of Lisbon approved the experimental protocol and materials prior to data collection. All participants gave written informed consent at the start of the experimental session.

### 2.3 Material and apparatus

Seventy‐two photorealistic scenes were created by placing target and distractor objects into a bare scene background. Of these, 24 were experimental scenes (crossing the four conditions described in the Design section), and 48 were fillers. To maintain comparability with previous results (Coco & Keller 2012), our analyses focus on eye movements and descriptions for the 24 experimental scenes. The scenes, data, and analysis scripts are available in the OSF archive for this paper.^1^

English speakers' eye‐movement data were acquired using an EyeLink II head‐mounted eye‐tracker (SR Research Ltd., Toronto, Canada) at 500 Hz. Images were displayed on a 21'' Multiscan monitor (1024×768 pixels), and speech was recorded via a lapel microphone. Data from Portuguese and Japanese speakers were acquired using an SMI IVIEW X HI‐SPEED tower‐mounted eye‐tracker (SensoMotoric Instruments, Teltow, Germany) at a sampling rate of 1250 Hz. Images were presented on a 19‐inch LG Flatron L194ws LCD screen (1920×1080 pixels), and speech was recorded using a standard desk‐mounted microphone. Only the dominant eye was tracked in both setups, determined by a simple parallax test.

### 2.4 Procedure

Each trial began with a cue word presented at the center of the screen for 750 ms. Immediately after, the scene appeared, and the sound recording was activated. Participants could view the scene for as long as needed to plan their description and were free to start speaking when ready. Upon completing their description, they pressed a button on a response pad to proceed to the next trial. The experiment was explained via written instructions, and participants were familiarized with the task through four practice trials. The session lasted approximately 45 min.

### 2.5 Similarity measures

This study examines whether semantic and syntactic information from sentences in three different languages predicts the similarity of associated scan patterns across two temporal windows: before and during speaking. This requires three distinct similarity measures: one for scan patterns, and two for the semantics and syntax of the associated sentences (see Fig. 1 for examples).

#### 2.5.1 Scan pattern similarity

Raw eye‐movement data were parsed into fixations and saccades using SR Data Viewer (English sample) or SMI BeGaze (Portuguese and Japanese samples). Both algorithms detect events based on velocity profiles of raw coordinates; default settings were applied in both cases.

To isolate the planning and articulation stages implied by the speech generation task, we divided fixations for each trial into two temporal segments: Before Speaking (from scene onset to utterance onset) and During Speaking (from utterance onset to utterance offset). On average, the pre‐speech inspection phase lasted 2.96 (±1.51) s for English speakers, 3.19 (±2.63) s for Japanese speakers, and 2.92 (±1.83) s for Portuguese speakers. The mean utterance duration was 3.38 (±2.46) s for English speakers, 5.06 (±3.37) s for Japanese speakers, and 8.13 (±8.42) s for Portuguese speakers.

Fixations in each phase were mapped to a categorical sequence of attended locations (scan pattern). We defined a 6×4 grid of cells of equal size over the scene. Fixations were assigned to consecutive 25 ms time bins labeled with the cell's categorical identifier (e.g., “aa,” see Fig. 1 for a visualization and refer to Coco (2022) for greater detail). We utilized the entire temporal sequence of fixations rather than restricting analysis to specific windows (e.g., around the verb) because our objective is to demonstrate that the full attentional motor output predicts concurrent linguistic output. Moreover, fixations are mapped against a regularly spaced grid rather than to specific target objects because all visual regions are potentially task‐relevant if participants deem them important for formulating their description.^2^

To measure similarity between scan patterns of varying lengths, we used the LCS algorithm (Gusfield, 1997). LCS is a dynamic programming technique that identifies the LCS between two sequences. The algorithm first creates a 2D matrix C with dimensions (m+1)×(n+1), where m and n are the lengths of the two sequences. It fills the matrix such that if the sequences match at indices i and j, C[i][j] is updated as C[i−1][j−1]+1. Otherwise, the cell takes the maximum value from its neighbors: C[i][j]=max(C[i−1][j],C[i][j−1]). For example, for sequences “ABCBDAB” and “BDCABC,” the LCS is “BCAB.” Similarity is then calculated as the ratio between the length of the LCS to the geometric mean of the lengths of the two sequences. To ensure robustness, we also computed similarities by mapping fixations to visual objects rather than grid cells, yielding nearly identical results (see Appendix A).

#### 2.5.2 Semantic similarity

To measure the semantic similarity between two sentences, we use the Universal Sentence Encoder (USE, Cer, Yang, Kong, Hua, Limtiaco, St. John, Constant, Guajardo‐Cespedes, Yuan, Tar, Strope & Kurzweil 2018), a transformer‐based language model that embeds sentences into a 512‐dimensional vector space. USE embeddings represent the distributional meaning of sentences, in analogy to how the meaning of words is defined by word embeddings such as word2vec (Mikolov, Sutskever, Chen, Corrado & Dean, 2013). We can then compare the semantic similarity of two sentences by computing the similarity of the two embedding vectors associated with the sentences. Several distance measures have been used in the literature; here, we will use the dot product, as cosine similarity yields identical results, given that they are positively correlated (r(2,830,891)=.98, p<.0001).

Crucially, USE embeddings are sensitive to word order, so the sentence embedding of *Peter likes his dog* and *his dog likes Peter* are different, reflecting their difference in semantics. Furthermore, USE embeddings are multilingual, so the USE model makes it possible to obtain embeddings for sentences in multiple languages from the same semantic space. Therefore, we can compare sentences such as *Peter likes his dog* and *O Peter gosta do seu cachorro* by computing the similarity between their USE vectors, even though the first sentence is in English, and the second one is in Portuguese. USE supports 12 languages, including English, Portuguese, and Japanese. We used the precomputed embedding models available with TensorFlow.^3^

#### 2.5.3 Syntactic similarity

To measure syntactic similarity while abstracting away from surface‐level lexical items, we utilized Ordered Dependency Tree Kernels. First, we parsed each sentence into a dependency tree using the SpaCy library.^4^ This parser utilizes a transition‐based approach, specifically a variant of the nonmonotonic arc‐eager transition system described by Honnibal & Johnson (2015), combined with pseudo‐projective dependency transformation proposed by Nivre (2005) to handle nonprojective structures. The parser achieves high accuracy, with labeled dependency accuracies of 0.94%, 0.86%, and 0.92% for English, Portuguese, and Japanese, respectively. We used the largest model available on the SpaCy website for each language.^5^

This process generates a hierarchical structure in which each word in a sentence is connected to its head, that is, to the word it syntactically depends on. The connecting arcs are labeled with the grammatical relation that holds between the head and its dependent (e.g., subject, object). For instance, the sentence *Peter likes his dog* is represented not just as a sequence of part‐of‐speech tags, but as a hierarchical structure, with *likes* as the root of the tree and *Peter* and *dog* as dependents of *likes*, labeled as subject and object, respectively. Examples of dependency structures from our data are shown in Fig. 1b.

The syntactic parsers we used produce trees consistent with the Universal Dependencies framework (de Marneffe, Manning, Nivre & Zeman, 2021). The aim of Universal Dependencies is to make syntactic structures comparable across languages, using a small inventory of dependency labels that apply to all languages. This is ideal for our purposes, as it enables the comparison of syntactic structures across languages, even if these languages are typologically distinct. The Universal Dependencies scheme has been successfully used to annotate 200 datasets across 150 languages.^6^

We then computed the similarity between dependency structures using a Subtree Kernel (specifically, the Subset Tree Kernel or SST), which counts the number of common substructures (fragments) shared between two trees (Moschitti et al., 2008). This method captures hierarchical syntactic information—such as the depth of embedding and the specific configuration of heads and dependents—that would not be captured if we compared only word sequences or part‐of‐speech sequences. Note that we configured the SST computation so that similarity is order‐dependent: for two trees to be considered similar, they must have the same head‐dependent relationships in the same order. This means that, for instance, SVO structures and OVS structures are not similar, even though they have the same head‐dependent relationships (V → S and V → O). Moreover, we removed both the words and part‐of‐speech labels from the dependency structures before computing their similarity, so as to capture syntactic, but not lexical, similarity.

To verify that our semantic and syntactic similarity measures capture distinct constructs, we calculated the Pearson correlation coefficients between them. We found extremely weak correlations both within (ρ=0.019,p<.0001) and across languages (ρ=−0.015,p<.0001). This reassures us that our similarity scores are effective at disentangling the contributions of syntactic and semantic information for predicting scan pattern similarity.

### 2.6 Syntactic diversity in the image descriptions

To quantify the cross‐linguistic diversity of the dependency structures in the participants' image descriptions, we computed descriptive statistics. Given that English, Portuguese, and Japanese are typologically different, we expect that word orders and dependency relations are not attested equally across the three languages. Testing this hypothesis is a way of validating our approach to computing syntactic similarity.

We took the dependency parses of the sentences in our dataset and removed all dependency labels except for the core dependencies. The core dependencies are the ones that indicate an obligatory grammatical relation between two words (i.e., modifiers are excluded). They are listed in Table 1. We keep the dependency labels in the order in which they occur in the parse, that is, they reflect the order of the words in the sentence. (Note that this is the order of the dependents, not the heads.) For example, for the dependency structure of s1 in Fig. 1b, the dependency label sequence would be “nsubj ROOT dobj,” while for the dependency structure of s2, it would be “nsubj ROOT nsubj dobj.”

**Table 1:** Core dependency labels used in the Universal Dependency annotation scheme

Label	Dependency
nsubj	nominal subject
nsubjpass	nominal subject of a passive clause
dobj	direct object
iobj	indirect object
csubj	clausal subject
csubjpass	clausal subject of a passive clause
ccomp	clausal complement
xcomp	open clausal complement
ROOT	root of the dependency tree

*Note*: that most of the utterances are either sentences, in which case, the ROOT is the main verb, or noun phrases, in which case, the ROOT is the head noun.

In Table 2, we tabulate the 10 most frequent sequences of core dependencies for each language. These sequences indicate word order patterns such as “nsubj ROOT” (SV; intransitive sentence in English) or “nsubj ROOT dobj” (SVO; transitive sentence in English). We observe substantial cross‐linguistic differences, which are consistent with our intuitions about syntactic differences between the three languages:

- In English, the main verb (with dependency label ROOT) is either preceded by a subject, or it is in first position. The latter case arises if the sentence uses a predicative construction (as in *there are two women in the room*). Moreover, a significant portion of the utterances in our data are noun phrases rather than sentences (as in *a room with two women*). In these cases, the head noun is the ROOT and also appears in first position. Note that additional subjects and objects can occur after the ROOT if embedded clauses are present (e.g., *a man jots notes on a clipboard while his friend watches* corresponds to “nsubj ROOT dobj nsubj”).
- In Portuguese, the picture is similar: the main verb is either preceded by a subject, or it is at the beginning of the sentence (as in *estão duas mulheres*, “there are two women”). Again, there are utterances that are noun phrases rather than sentences, which also means the ROOT (the head noun) is in first position. In Portuguese, we also observe post‐verbal complement clauses, with dependency label xcomp. (These also occur in English, but not among the 10 most frequent structures.)
- The contrast with Japanese is striking: Here, all the ROOTs of dependency structures (main verbs and head nouns) are in final position, consistent with the fact that Japanese is a head‐final language. (In fact, ROOT is in the final position in all the dependency structures in our Japanese utterances data, not just in the 10 most frequent ones.) As in English and Portuguese, we observe structures with multiple subjects and objects, which indicate subordinate clauses. But in Japanese, all of them occur before the ROOT.

Taken together, we observe that there are clear syntactic differences between the languages: in Japanese, the head of an utterance is always in final position, whereas in English and Portuguese, the head of an utterance can occur in either first or second position. First position is more frequent in Portuguese than in English, perhaps due to the greater prevalence of predicative constructions in that language. This demonstrates that our approach which computes syntactic structures using universal dependencies is able to pick up grammatical differences between languages, as expected.^7^

**Table 2:** The most frequent sequences of core dependency labels across the three languages. These patterns indicate typological differences in word order. ROOT marks the head of a dependency structure

English		Portuguese		Japanese	
#	Word order	#	Word order	#	Word order
198	nsubj ROOT	160	ROOT	175	nsubj ROOT
96	nsubj ROOT dobj	77	ROOT dobj	94	nsubj dobj ROOT
88	ROOT	77	nsubj ROOT	35	nsubj nsubj ROOT
37	ROOT dobj	36	nsubj ROOT xcomp dobj	18	nsubj nsubj dobj ROOT
21	nsubj ROOT nsubj	25	nsubj ROOT xcomp	17	nsubj nsubj nsubj ROOT
15	nsubj ROOT dobj nsubj	22	nsubj ROOT dobj	15	dobj nsubj ROOT
14	nsubjpass ROOT	14	ROOT nsubj	14	ROOT
8	ROOT nsubj dobj	13	ROOT dobj dobj	14	nsubj dobj dobj ROOT
7	ROOT nsubj	9	ROOT nsubj dobj	14	dobj ROOT
7	nsubj ROOT dobj nsubj dobj	8	ROOT xcomp dobj	13	nsubj dobj nsubj ROOT

### 2.7 Data processing and statistical inference

For each participant (N=74), we considered the 24 sentences and associated scan patterns generated for the experimental trials, which gives us a total of 1776 trials (74×24). Of those unique trials, we excluded 36 sentences that were either too short or too long (i.e., the bottom 2% and top 99% of their distribution, corresponding to sentences with fewer than four words or longer than 77 words) and five further sentences because the associated audio file was corrupted. Regarding the scan patterns, we removed those with fewer than two fixations within the selected temporal window of interest, indicating poor‐quality data (i.e., 12 before speech and 91 during speech). Similarities are computed pairwise: every sentence is compared with every other sentence, and likewise, each scan pattern is compared with every other scan pattern, independently for each of the two attentional phases considered (before and during speech). This calculation yielded a total of N=2,830,893 data points for the analysis. Statistical inference used the linear‐mixed‐effects modeling framework implemented in the lme4 package in R (Bates, Mächler, Bolker & Walker, 2015). Our dependent variable is scan pattern similarity, which we predict in a single model as a function of (a) the semantic and syntactic similarity of sentences, (b) whether the similarity score is within (or between) scenes (with between as the reference level), (c) whether the similarity score comes from comparing sentences (or scan pattern) of the same (or different) language (with different as the reference level), and (d) the speech production for which the similarity score was computed, that is, before or during speaking (with before as the reference level).

We also tested what happens to the association between scan pattern and sentence similarity when we shuffle the data points, that is, we randomly pair each scan pattern with a sentence rather than the one it occurred with. This control analysis assesses whether the association between a sentence's semantic and syntactic information actually modulates scan‐pattern similarity, rather than being an artifact independent of the scan pattern with which a sentence is paired. In the Supplementary Material, we report the full analysis for the shuffle condition, which shows that the effect of semantic and syntactic similarity is now absent. We also include the shuffle condition as a baseline in our visualizations (gray line).

The random effects considered in our models are Pairs of Participants (N=2775), Pairs of Items (N=4656), and Pairs of Cue Words ^8^ (N=435). We build random‐intercept‐only models with a full fixed‐effect structure, that is, all possible main effects and interactions are included. We are aware that modeling only random‐intercept models may be anticonservative, and that maximal‐random models (Barr, Levy, Scheepers & Tily, 2013) would be preferable in certain contexts. However, a model of that form does not converge, and it would be overparametrized, considering that we are including five fixed effects and three random effects. ^9^ Moreover, when plotting the model‐predicted values against the raw data, we find a very close correspondence, demonstrating that a random‐intercept model fully captures the variability in our data (see Fig. 4). In Table 3, we report the coefficients of the final model (also standardized), its confidence intervals, which can be used to reconstruct the size and reliability of the estimates (Luke, 2017), its t‐values and p‐values based on asymptotic Wald tests, with asterisks indicating their level of significance (e.g., ∗:p<.05).

**Table 3:** Linear mixed‐effects model for predicting the similarity of two scan patterns as a function of Semantics (semantic similarity between the sentences associated with the scan patterns; dot‐product over sentence embeddings), Syntax (tree kernel similarity between syntactic dependency trees), Language (sentences use the same or a different language; different as reference Level), Scene (sentences describe the same or a different visual scene; different as reference level), and Phase (the temporal segments in the production task, distinguishing between before and during speaking; before as reference level). The participant pair (n=2775), item pair (n=4656), and word cue pair (n=435) were the random variables introduced as intercepts

Predictors	β (Std. β)	CI (2.5 %; 97.5 %)	*t*‐value^10^
(Intercept)	0.183 (1.534)	0.179; 0.187	84.39^***^
Semantics	−0.002 (−0.003)	−0.006; 0.001	−1.29
Syntax	0.002 (0.002)	−0.001; 0.004	1.21
Language	0.022 (0.087)	0.019; 0.025	15.49^***^
Scene	0.112 (0.133)	0.101; 0.123	20.05^***^
Phase	−0.026 (−0.109)	−0.027; −0.025	−45.17^***^
Semantics x Syntax	0.068 (0.037)	0.057; 0.079	12.02^***^
Semantics x Language	−0.013 (−0.014)	−0.019; −0.007	−4.52^***^
Language x Syntax	0.027 (0.049)	0.023; 0.031	14.03^***^
Semantics x Scene	0.022 (0.012)	0.004; 0.040	2.39^*^
Scene x Syntax	−0.022 (−0.009)	−0.044; 0.001	−1.90
Language x Scene	−0.006 (−0.004)	−0.020; 0.007	−0.97
Semantics x Phase	0.094 (0.106)	0.089; 0.100	36.57^***^
Syntax x Phase	0.001 (0.001)	−0.003; 0.004	0.30
Language x Phase	−0.006 (−0.017)	−0.008; −0.004	−5.68^***^
Scene x Phase	−0.082 (−0.067)	−0.093; −0.071	−14.53^***^
Semantics x Language x Syntax	−0.016 (−0.008)	−0.031; −0.002	−2.21^*^
Semantics x Scene x Syntax	0.128 (0.027)	0.077; 0.179	4.92^***^
Semantics x Language x Scene	0.041 (0.013)	0.013; 0.068	2.87^**^
Language x Scene x Syntax	0.046 (0.013)	0.014; 0.078	2.81^**^
Semantics x Syntax x Phase	−0.218 (−0.098)	−0.234; −0.202	−27.22^***^
Semantics x Language x Phase	0.033 (0.026)	0.025; 0.040	8.19^***^
Language x Syntax x Phase	−0.072 (−0.098)	−0.077; −0.067	−27.93^***^
Semantics x Scene x Phase	0.015 (0.006)	−0.011; 0.040	1.15
Scene x Syntax x Phase	0.095 (0.028)	0.062; 0.127	5.76^***^
Language x Scene x Phase	0.001 (0.001)	−0.018; 0.020	0.13
Semantics x Language x Scene x Syntax	−0.124 (−0.019)	−0.191; −0.058	−3.67^***^
Semantics x Language x Syntax x Phase	0.086 (0.031)	0.065; 0.106	8.19^***^
Semantics x Scene x Syntax x Phase	−0.109 (−0.015)	−0.182; −0.035	−2.89^**^
Semantics x Language x Scene x Phase	−0.046 (−0.011)	−0.086; −0.007	−2.28^*^
Language x Scene x Syntax x Phase	−0.050 (−0.010)	−0.097; −0.004	−2.12^*^
Semantics x Language x Scene x Syntax x Phase	0.107 (0.011)	0.010; 0.203	2.17^*^

*Note*: $p<.001^{***}$; $p<.01^{**};p<.05^{*}$

**Fig. 2:**
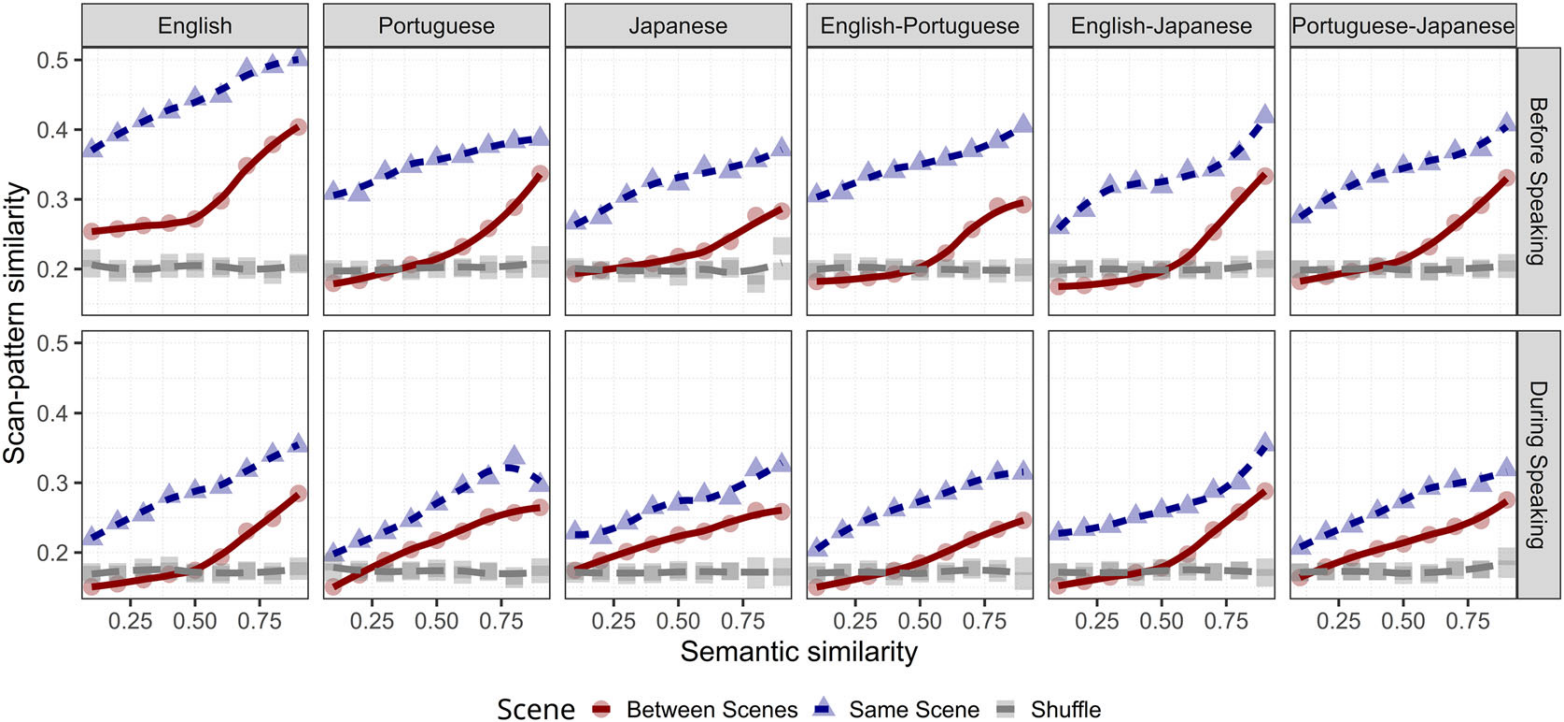
Scan pattern similarity as a function of semantic similarity (with dot‐product over sentence embeddings as similarity measure). Within each panel, we compute the mean of scan pattern similarity (y‐axis) over regularly spaced bins of semantic similarity (x‐axis, ranging from 0 to 1 in increments of 0.1) and indicate the trend of the mean (as a loess line). Line color, font, and symbol type indicate whether the similarities have been computed within the same scene (blue triangle), between different scenes (red circle), or in the shuffled condition (gray square). All comparisons between the different languages are displayed across the panels (English; Portuguese; Japanese; English–Portuguese; English–Japanese; Portuguese–Japanese), while Phases are compared as rows, with top panels showing similarities computed before speaking, while the bottom panels show similarities during speaking.

**Fig. 3:**
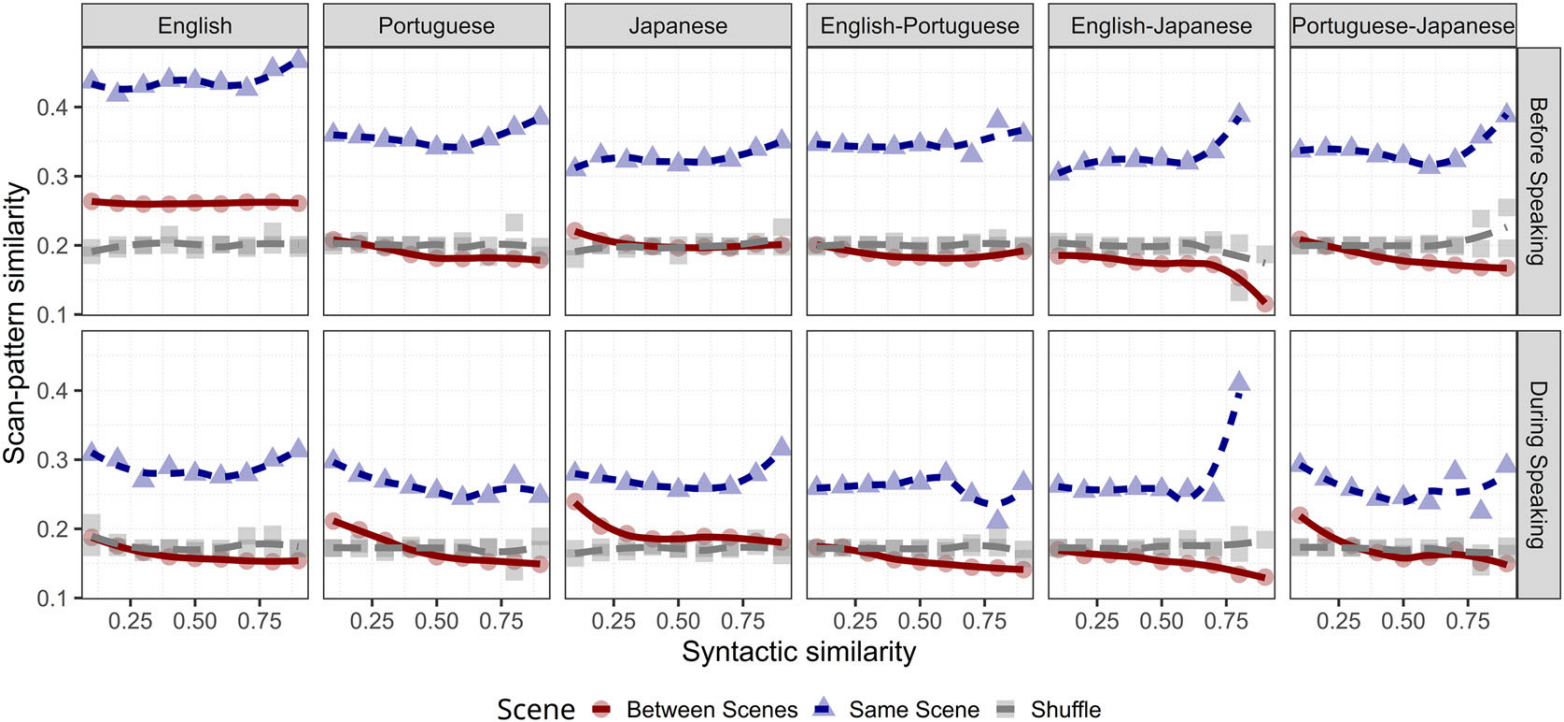
Scan pattern similarity as a function of syntactic similarity (with ordered tree kernel as similarity measure). Within each panel, we compute the mean of scan pattern similarity (y‐axis) over regularly spaced bins of syntactic similarity (x‐axis, ranging from 0 to 1 in increments of 0.1) and indicate the trend of the mean (as a loess line). Line color, font, and symbol type indicate whether the similarities have been computed within the same scene (blue triangle), between different scenes (red circle), or in the shuffled condition (gray square). All comparisons between the different languages are displayed across the panels (English; Portuguese; Japanese; English–Portuguese; English–Japanese; Portuguese–Japanese), while Phases are compared as rows, with top panels showing similarities computed before speaking, while the bottom panels show similarities during speaking.

**Fig. 4:**
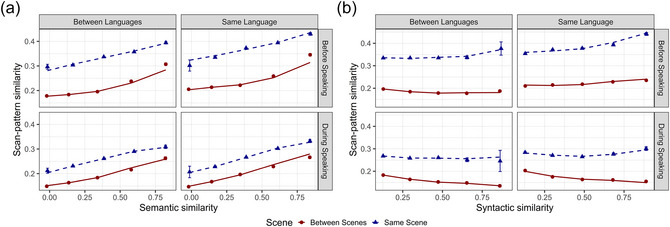
Visualization of conditional model prediction and observed (raw) scan‐pattern similarity for the two four‐way interactions of Language, Phase, Scene, and Semantics, panel (A) or Syntax, panel (B). Within each panel, the mean of scan pattern similarity (y‐axis) is computed over regularly spaced bins of semantic or syntactic similarity (x‐axis, ranging from 0 to 1 in increments of 0.1) and overlaid by the model‐predicted fit (as a line). Languages are organized as columns in the panels and aggregated into between‐language and same‐language comparisons. The Phases of the task, before and during speech, are instead organized as rows. Line color, font, and symbol type indicate whether the similarities have been computed within the same scene (blue triangle) and between different scenes (red circle).

## 3 Results

In this study, we eye‐tracked participants as they described cued objects embedded in visual scenes in one of three languages (English, Portuguese, or Japanese). The core objective was to investigate whether the semantic and syntactic constraints in sentences guide the allocation of overt attention, and to discern the roles of these representational systems during two critical phases of the speech generation process: the planning phase (before speaking) and the articulation phase (during speaking). Pairwise similarity was computed between all descriptions—both within and across languages, and for the same or different scenes. This design allows us to test the extent to which effects transcend specific linguistic constraints (e.g., Japanese SOV word order vs. English SVO) and visual contexts. Semantic similarity was computed using neural sentence embeddings (Cer et al., 2018), which enable the comparison of sentence meanings using high‐dimensional vector representations that abstract away from differences in syntactic form. Syntactic sentence similarity was computed as the tree kernel similarity of dependency representations, capturing hierarchical relationships between syntactic constituents. Finally, the similarity between the scan patterns associated with the sentence was computed using LCS sequence comparison across the two phases. We fitted a statistical model that predicts the similarity of two scan patterns based on the interactions between Semantics (semantic similarity between the two sentences), Syntax (syntactic similarity between the two sentences), Language (Same vs. Different language), Scene (Same vs. Different visual scene), and Phase (Before vs. During speaking). We primarily discuss our results using visualizations (see Figs. 2, 3, 4) and focus on the effects of theoretical interest (see Table 3 for the full model output).

### 3.1 Cross‐modal coordination is primarily driven by semantic similarity within and between languages throughout the entire speech generation process

Fig. 2 graphs semantic sentence similarity (x‐axis) against scan‐pattern similarity (y‐axis). As confirmed by the model coefficients in Table 3, semantic similarity is a significant predictor of scan pattern similarity. Crucially, this finding holds not only for all three languages (English, Portuguese, and Japanese) but also across all between‐language comparisons (English–Portuguese, English–Japanese, Portuguese–Japanese). Intuitively, this means that if a Japanese speaker utters a sentence that is semantically similar to one spoken by an English speaker, they follow similar scan patterns. We thus not only replicate prior results for English (Coco & Keller 2012) but generalize this finding to languages with distinct syntactic structures: Japanese is a verb‐final language, whereas English is verb‐medial; English is modifier‐first, whereas Portuguese is modifier‐final.

Another remarkable result is that semantic similarity predicts scan pattern similarity both when the message is planned and when it is articulated. This finding is consistent with the existence of a prelinguistic LoV acting as a stable representational system that samples visual information from the scene and later linearizes it into a structured utterance; such a compositional mechanism is active throughout. More critically, the LoV representational system appears to be universal within and across languages, as evidenced by the model predictions in Fig. 4 (Panel A), which show near‐parallel same‐ and between‐language regression lines. Additionally, this effect holds when comparing sentences produced for the same visual scene (blue triangles) and for different visual scenes (red circles). While similarity is naturally lower in the between‐scene condition, the positive association remains clearly visible. This suggests that the low‐level perceptual qualities of visual scenes have a comparatively smaller impact on overt attention than the guidance provided by their high‐level semantic content.

### 3.2 Cross‐modal coordination is modulated by syntactic similarity within the same language and scene context, primarily before speaking

The results in Fig. 2 and Fig. 4 (Panel A) strongly suggest that scan patterns and sentences are associated through semantic similarity across languages and scenes throughout the entire speech generation process. However, syntactic constraints could also play a role. To elucidate this, Fig. 3 plots the syntactic similarity of sentences (x‐axis) as a predictor of scan pattern similarity (y‐axis). Recall that syntactic information is represented as dependency trees, which express the structural relationships between heads and their dependents. Therefore, our measure of similarity, based on the number of shared substructures, captures structural complexity beyond simple word order. We also showed that our measures of semantic and syntactic similarity are uncorrelated.

Examining the three languages and their pairings in Fig. 3, we observe a much weaker effect of syntactic compared to semantic constraints. In particular, we find no clear association for the between‐scene condition (red lines), where the relationship is almost identical to the baseline (shuffling, i.e., randomized association)—if anything, the red lines show a small negative association. Furthermore, even in the within‐scene condition (blue lines), the effect is mainly present when comparing sentences of the same language (Fig. 4, Panel B, right) rather than sentences of different languages (left).

Crucially, the effect of syntax is not only localized within language and scene context but is also temporally locked to the planning phase of the utterance (i.e., before speaking). Syntactic similarity is associated with similar scan patterns primarily in the prespeaking (planning) phase; during the articulation phase, this effect diminishes or reverses for between‐scene comparisons. This finding highlights a localized, context‐dependent role for syntactic constraints and specifies the conditions under which the syntax–semantics interface is most active. The restriction of syntactic effects to the planning phase confirms that while syntactic information acts as a constraint during structural planning and message linearization, semantic information maintains primacy in guiding eye movements once articulation begins.

To ensure that the observed associations were not due to chance or artifacts—such as center bias (Tatler, 2007) or intrinsic scene properties—we performed a control analysis by shuffling the correspondence between scan patterns and sentences. By disrupting the specific link between a scan pattern and its associated utterance, the significant effect of semantic similarity disappears (illustrated by the gray lines in Fig. 2 and confirmed by inferential statistics in the Supplementary Material, S1). Moreover, we largely corroborate these results even when syntax is more coarsely represented as a sequence of parts of speech, and similarity is computed using the LCS algorithm (refer to Supplementary Material, S2, for the output of this analysis). Finally, we confirmed that these results are robust regardless of whether fixations are mapped against a rectangular grid or to precise visual objects (see Appendix A).

Overall, these results suggest that the association between scan patterns and sentences is primarily predicted by semantic similarity, reflecting the stable scaffolding of a universal LoV. Syntactic constraints play a secondary role, confined to the early planning phase of the utterance and to pairs where both the visual scene and the language are identical.

## 4 Discussion

This study provided empirical evidence that the similarity of participants' scan patterns is predicted by the semantic similarity of the associated scene descriptions across typologically diverse languages (English, Portuguese, and Japanese). Our results extend previous work (Coco & Keller 2012) to languages beyond English. As discussed in the Introduction, our findings enable us to adjudicate between the LoV hypothesis (Cavanagh, 2021), a strictly syntactic hypothesis, and interactionist accounts regarding the representational systems that coordinate overt attention and speech production.

The idea of an LoV builds on the LoT hypothesis (Fodor, 1975) in assuming the semantic compositionality of prelinguistic representations. Our results support this hypothesis and suggest that the relevant semantic representations are shared across languages and grounded in the perceptual system (Wilson et al., 2022; Quilty‐Dunn et al., 2023). This is also consistent with the idea that visual information shapes the language used to describe it (Talmy, 1975; Landau & Jackendoff, 1993; Hafri et al., 2022; Ünal et al., 2024; Coventry et al., 2023) and with recent evidence showing that speakers consistently prioritize affordance‐based object semantics and organize visual scenes into meaningful conceptual clusters as they incrementally build utterances (Barker, Rehrig, & Ferreira, 2023; Tachihara, Barker, Cotter, Hayes, Henderson, Zhou & Ferreira, 2026). While these associations are robust, it remains possible that such semantic primacy reflects a general cognitive structuring of visual information, a “grammar of the scene,” that operates whenever a task requires meaningful extraction, rather than being driven solely by the language system itself (see Murlidaran & Eckstein (2025) for recent evidence from a free‐viewing task). Furthermore, overt attention, as a motor output of the visual system, encodes grammatical cues of the compositional processes that occur when the acquisition of visual information simultaneously engages the human sentence processor (Gregory, 1974; Cavanagh, 2021; Webb, Knott & MacAskill, 2010). Lastly, the evidence of cross‐linguistic universality of an LoV, and especially its dominant role throughout the entire speech generation process (i.e., before and during speaking), raises new questions about the interface between semantics and syntax, requiring novel paradigms beyond the logical or experimental approaches commonly adopted (e.g., Friederici & Weissenborn (2007); Hackl (2013); see Morgan, van der Meer, Vulchanova, Blasi & Baggio (2020) for a review).

In contrast to predictions about an LoV, a strictly syntactic hypothesis holds that the grammatical structure of utterances drives coordination with overt attention. This hypothesis, however, received limited support in our study. Syntactic similarity predicted scan patterns within scenes for sentences in the same language, but this effect was absent between scenes (if not negative) and significantly attenuated across languages. Note that we computed syntactic structure similarity using dependency trees, which are inherently hierarchical and can capture nonadjacent relationships between syntactic constituents. This finding suggests that the limited role of syntax in predicting scan pattern alignment is not due to a shallow representation of syntactic information (see the Supplementary Material, S2, for alternative analyses that utilize sequences of part‐of‐speech tags instead of dependency trees, yielding broadly similar results).

Even if the effect of syntax was much weaker than semantics in coordinating with attentional processes, our results still indicated a nuanced influence. When we separated the time course of speech generation into an early planning phase (before speech onset) and a late delivery phase (during speech), we found that syntactic constraints were primarily active during the early phase, particularly in relation to within‐language and within‐scene comparisons. While the visual system extracts a global, nonlinear event structure (Zacks & Tversky, 2001), the language system must impose a linear order on these relations (Gleitman et al., 2007; Papafragou et al., 2008; Myachykov et al., 2011). Consequently, syntactic constraints such as those dictated by word order or thematic role assignment are most critical during this structural formulation phase, where nonlinear meaning is converted into a linear sequence (Lashleyet al., 1951; Dell et al., 1997). This restriction likely reflects the allocation of limited attentional and working memory resources, as generating a sentence requires holding a linear sequence of grammatical slots in active memory (Chen, Qiu & Wang, 2025; Huettig, Olivers & Hartsuiker, 2011; Souza & Skóra, 2017). Because detailed syntactic planning is computationally expensive, the system minimizes this load by relying on the LoV as a form of stable external memory. Syntax is thus accessed transiently only during the structural formulation (early) phase, before the system offloads guidance back to global semantics during the articulation phase (Levelt et al., 1999). These findings suggest a representational hierarchy where semantics provides the stable, nonlinguistic scaffolding, while syntax fine‐tunes local attentional responses based on language and context—especially when the message must be formulated for output. As these adjustments likely occur at the syntax–semantics interface, they lend empirical support to an interactive architecture of cross‐modal integration (Myachykov et al., 2007; Jackendoff, 1992, 2025). However, they seem to play a secondary role compared to the initial meaning representation provided by a prelinguistic LoV (see Poletiek, Monaghan, van de Velde & Bocanegra (2021) for evidence on artificial language learning).

Admittedly, our study is inherently correlational; while linguistic similarity scores are powerful predictors of scan‐path alignment, the lack of direct experimental manipulation of these variables precludes definitive causal claims. This localized effect of syntax may reflect a key methodological distinction between our speech generation task—where planning and articulating lead to an inherently variable time‐course—and classic studies in the visual world paradigm (e.g., Tanenhaus et al. 1995; Altmann & Kamide 1999; Spivey, Tanenhaus, Eberhard & Sedivy 2002) that typically evaluate language comprehension of specific syntactic constructions, allowing precise time‐locking of anticipatory eye movements to incremental, word‐by‐word processes. Scene descriptions in our study did not tap into precise compositional processes, such as symmetric relationships (Hafri et al., 2022), thematic role assignment in agentive verbs (Koring, Mak & Reuland, 2012), or ergative verbs, which emphasize the object role in an action (see Khatin‐Zadeh, Hu, Eskandari, Banaruee, Yanjiao, Farsani & He (2024) for an example of gestures). Furthermore, since production and comprehension are not distinct and likely share predictive forward models (e.g., Kempen, Olsthoorn & Sprenger 2012; Pickering & Gambi 2018), a visual world version of our study may be able to better elucidate the moment‐by‐moment online integration of overt attention and sentence understanding (Snedeker & Trueswell, 2004), and thus examine whether the evidence of semantic dominance observed in our data still holds. Future research could, for example, develop experimental designs that elicit descriptions involving actions, agents, and patients across languages, yielding a more comprehensive understanding of the LoV underpinning event representation and its reflection in overt attention. Additionally, by systematically manipulating syntactic constructions in their complexity, it is plausible to map their cartographic distances as a function of gaze alignment. While we expect semantic similarity to remain dominant, this controlled setting could isolate structural distinctions linked to predictable gaze shifts, effectively testing the comprehension–production interface (Pickering & Gambi, 2018). The key question here will be whether the metrics that constrain a producer's prespeech attention also predict a comprehender's referential target. Furthermore, by manipulating the position of crucial linguistic cues (e.g., late vs. early verb), we may be able to map divergence in online structural prediction, directly testing how the timing of such cues interacts with the universal event structure (LoV) rapidly extracted from visual input (Hafri & Trueswell, 2025), thereby contributing to the debate about a unified, predictive framework for both language modalities.

Another critical aspect of our results is that they rely entirely on oculomotor behavior. However, to determine at a finer temporal resolution which aspects of sequential visual processing predict semantic composition and how these processes may diverge across languages during syntactic phrase building, it is necessary to integrate eye movements with electrophysiological responses. This approach would allow us to map the electrophysiological indices assumed to underlie phrase‐building operations (see Maran, Friederici & Zaccarella (2022) for a recent review) against concurrently processed chunks of visual information. A consistent body of evidence demonstrates that electrophysiological activity directly maps onto overt attention in tasks such as reading, visual search, or short‐term memory during natural viewing behavior (e.g., Coco et al. 2020; Dimigen, Kliegl & Sommer 2012; Kaunitz, Kamienkowski, Varatharajah, Sigman, Quiroga & Ison 2014). Therefore, components in neural activity indicating the semantic (e.g., the N400) or structural (e.g., the P600) predictability of visual information (Coco et al., 2017; Lauer et al., 2018; Mudrik et al., 2010; Mudrik et al., 2014; Võ & Wolfe, 2013; Wolfe et al., 2011) may be concurrently mapped onto language processes to define more precisely the characteristics of an LoV or scene grammar (Võ, 2021; Võ et al., 2019).

A key aspect of our study was the utilization of linguistic embeddings. Word embeddings are dense vectors trained to represent the contextual properties of words (Mikolov et al. 2013). The embeddings of similar words (in terms of which other words they co‐occur with) are close together in vector space. Therefore, measures such as the dot product or cosine between embedding vectors can be used to compute word similarity. Embeddings have been generalized from words to sentences, and multilingual embedding models have been developed accordingly. In this study, we deployed the USE (Cer et al., 2018), which enabled us to compare the meanings of sentences across different languages, abstracting away from their syntactic realizations. While we make no claims about the cognitive plausibility of the specific sentence embedding model we have employed, we would like to note that linguistic embeddings have a long history as models of human language processing (for an overview, see Merkx, Frank & Ernestus 2022). More generally, neural networks, typically the modeling approach used to compute word or sentence embeddings, are a classic tool in cognitive science, going back at least to the Parallel Distributed Processing framework (McClelland, Rumelhart, Group & others, 1987). On the other hand, neural network models have been criticized because they can be uninterpretable “black boxes,” not constrained by biological realism, engineered to optimize performance in specific tasks, and requiring learning regimes that are unrealistic compared to humans (see Bowers, Malhotra, Dujmović, Montero, Tsvetkov, Biscione, Puebla, Adolfi, Hummel, Heaton, & others (2023) for a discussion focused on the visual system). Yet, other researchers disagree with these statements, instead advocating for neural nets as credible models of human cognitive processes, including vision (Kriegeskorte, 2015; Cichy & Kaiser, 2019), memory (Heinen, Bierbrauer, Wolf & Axmacher, 2024), and linguistic processing (Piantadosi, 2023; Hahn & Keller, 2023).

Computationally, the current study did not directly investigate real‐time alignment between visual and linguistic processing; instead, we focused on the association between the two information streams, using sentence similarity as a predictor of scan pattern similarity. However, our reliance on global similarity metrics may overlook fine‐grained individual differences in attentional strategies. Future research could benefit from network‐based scan‐path analyses, where eye movements are modeled as transition networks to reveal structural metrics (e.g., centrality, density) capable of distinguishing processing styles (Ma, Liu, Clariana, Gu & Li, 2023). Additionally, recent advances in deep semantic gaze embeddings (Castner, Kuebler, Scheiter, Richter, Eder, Hüttig, Keutel & Kasneci, 2020) and personalized scan‐path prediction models using observer encoders (Chen, Jiang & Zhao, 2024; Xue, Xu, Mondal, Le, Zelinsky, Hoai & Samaras, 2025) may offer higher granularity in distinguishing between participants who rely on top‐down linguistic guidance versus those driven by bottom‐up visual saliency. In parallel, the past decade has seen rapid advances in representing visual information using formalisms previously applied to linguistic information (e.g., dependency grammars, Elliott & Keller 2013), leading to new developments in multimodal modeling (Koh, Fried & Salakhutdinov, 2023), with applications to tasks such as image captioning (Elliott & de Vries, 2015), text‐to‐image generation (Rombach, Blattmann, Lorenz, Esser & Ommer, 2022), or sign language recognition (Li, Duan, Fang, Gong & Jiang, 2020) (see Wu, Gan, Chen, Wan & Philip (2023) for a review of state of the art).

Moreover, the range of languages investigated in our study needs to be expanded. In follow‐up work, examining a more comprehensive array of languages, especially those with diverse linguistic features, would be essential to strengthen the cross‐linguistic validity of our findings. Linguistic diversity is also a heavily debated topic, with its core rooted in the Sapir–Whorf hypothesis, positing that language plays a role in structuring our thoughts (Kay & Kempton, 1984; Gentner, 2003; Wolff & Holmes, 2011). Our findings indicate that the connection between thought processes and perception may be more robust than the connection between thought and language (Levinson, 1997; Hespos & Spelke, 2004). However, this does not rule out the possibility that language plays a critical role in shaping thought, particularly in fundamental domains like space, where language may actively restructure cognition (Papafragou et al., 2008; Gennari, Sloman, Malt & Fitch, 2002; Majid, Bowerman, Kita, Haun & Levinson, 2004; Davidoff, Davies & Roberson, 1999). This influence of language on perception may be dynamic rather than static, with lexically specific labels acting as top‐down cues that rapidly, but transiently, modulate visual discrimination (Lupyan, Rahman, Boroditsky & Clark, 2020). Furthermore, language per se also entails other communicative mechanisms, such as co‐speech gestures, which differ cross‐culturally and may serve to convey specific frames of reference (Ünal, Mamus & Özyürek, 2024). Consequently, more research is needed to investigate languages with distinct grammatical structures (see Haspelmath, Dryer, Gil & Comrie (2008) for a world atlas), and communicative nonlinguistic acts more broadly, to shed light on the impact of culturally specific experiences on the development and expression of the LoV.

Altogether, our study suggests that the visual system employs structured meaning representations, as predicted by the LoT hypothesis, and that these representations can be interpreted through patterns of overt attention. These patterns are shared across languages, even when their surface realizations differ. This finding has a significant impact on our understanding of the compositional mechanisms underlying cognitive processing across multiple modalities.

## Supporting information


**Appendix S1**:Internet Appendix.

## Notes

1
https://doi.org/10.17605/OSF.IO/6USMK

2 We did not define areas of interest that are pertinent to our experimental hypothesis. However, for completeness, here are the statistics for the two visual objects referring to the cue word: Animate objects were viewed more quickly (1.93 ±2.38 s) and for slightly less time (317.67 ±262.45 ms) than inanimate objects (2.30 ±2.41 s; 334.14 ±268.46 ms).

3
https://www.kaggle.com/models/google/universal‐sentence‐encoder

4 The models are available from https://spacy.io/models/.

5
https://spacy.io/models/en#en_core_web_trf, https://spacy.io/models/pt#pt_core_news_lg, https://spacy.io/models/ja#ja_core_news_trf

6 See https://universaldependencies.org/ for more details.

7 Another example is the placement of adjectival modifiers of nouns: These occur prenominally in English and Japanese, but postnominally in Portuguese. Sentential modifiers of nouns (relative clauses, for example), on the other hand, occur prenominally in Japanese, but postnominally in English and Portuguese. These differences are also attested in our data, but the details are omitted here for brevity.

8 We are considering 96 items, rather than 24 scenes, to capture the variability arising from the animacy of the cue (animate, inanimate), and clutter of the scene (minimal, cluttered). The total number of cue words is 29, rather than 48, because some cues were repeated (e.g., man or woman). Refer to the OSF repository for the full list of cue words used in this study.

9 We also did not want to apply an automatic forward (or backward) selection procedure (Kuznetsova, Brockhoff & Christensen, 2017), because the parameters retained in the final model may be arbitrarily selected.
