# Supplementary material for: The Cross‐Linguistic Coordination of Overt Attention and Speech Production as Evidence for a Language of Vision
Source: Cogn Sci. 2026 Feb 23;50(2):e70185. doi: 10.1111/cogs.70185 (PMC12930141; doi:10.1111/cogs.70185)
Supplement: Supplementary file 1 — Appendix S1:Internet Appendix. [file COGS-50-e70185-s001.docx]

**Supplemental Material**

for

**The Cross-linguistic Coordination of Overt Attention and Speech Production as Evidence for a Language of Vision**

Moreno I. Coco^1,2^, Eunice G. Fernandes^3^, Manabu Arai^4^ & Frank Keller^5^

^1^ Department of Psychology, Sapienza University of Rome, Rome, Italy

^2^ IRCCS Fondazione Santa Lucia, Rome, Italy

^3^ School of Psychology, University of Minho, Braga, Portugal

^4^ Faculty of Economics, Seijo University, Tokyo, Japan

^5^ School of Informatics, University of Edinburgh, Edinburgh, United Kingdom

* Correspondence about this work can be addressed to Moreno I. Coco ([moreno.coco@uniroma1.it](mailto:moreno.coco@uniroma1.it)) or Frank Keller ([keller@ed.ac.uk](mailto:keller@ed.ac.uk))

**Supplemental Material S1**

*Shuffling the association between scan patterns and sentences disrupts semantic and syntactic effects.*

To confirm that the observed relationship between linguistic information (semantic and syntactic) and visual attention is genuine—and not a statistical artefact of our similarity metrics—we performed a control analysis. We disrupted the functional link between modalities by randomly shuffling the data points. Crucially, this shuffling was performed independently within each phase: each scan pattern was paired with a randomly selected sentence from the same temporal window (i.e., pre-speaking scan patterns were shuffled only with other pre-speaking trials, and likewise for the speaking phase). If the effects reported in the main text are driven by the specific coupling of speech and gaze, they should vanish in this shuffled dataset. As illustrated by the flat grey lines in Figures 2 and 3 of the main manuscript, the shuffled condition shows no modulation by semantic or syntactic similarity. The model output (Table S1) confirms this observation: neither semantic nor syntactic similarity significantly predicts scan pattern similarity in the shuffled data. The only significant predictor remaining in the model is Phase. We observed lower scan pattern similarity during the speaking phase compared to the pre-speaking phase. Because the shuffling was restricted within each phase, the inherent distributional differences between the two temporal windows were preserved. Specifically, scan patterns are naturally more variable (and thus less similar on average) during the longer, more complex articulation phase compared to the initial planning phase. Since we did not mix data across phases, this baseline difference remains significant. Crucially, no other main effects or interactions were significant. This analysis confirms that the coordination between linguistic content and overt attention observed in the main analysis is not an artefact, but reflects a true functional coupling between the two modalities.

**Table S1:** Linear mixed-effects model for predicting the similarity of two scan patterns (after their association with sentences is randomly shuffled) as a function of Semantics (semantic similarity between the sentences associated with the scan patterns; dot-product over sentence embeddings), Syntax (tree kernel similarity between syntactic dependency trees), Language (sentences use the same or a different language; different as reference Level), Scene (sentences describe the same or a different visual scene; different as reference level) and Phase (the temporal segments in the production task, distinguishing between before and during speaking; before as reference level). The participant pair (N = 2,775), item pair (N = 4,656), and word cue pair (N = 425) were the random variables introduced as intercepts.

| Predictor | $\beta$ (Std. $\beta$) | CI (2.5 %; 97.5 %) | t-value |  |
| --- | --- | --- | --- | --- |
| (Intercept) | 0.200 (1.681) | 0.199; 0.201 | 426.51*** |  |
| Semantics | -0.002 (-0.002) | -0.006; 0.002 | -0.90 |  |
| Syntax | -0.000 (-0.001) | -0.003; 0.002 | -0.26 |  |
| Language | -0.001 (-0.004) | -0.002; 0.001 | -1.13 |  |
| Scene | -0.005 (-0.006) | -0.014; 0.004 | -1.13 |  |
| Phase | -0.028 (-0.118) | -0.029; -0.027 | -41.53*** |  |
| Semantics x Syntax | 0.003 (0.001) | -0.010; 0.015 | 0.41 |  |
| Semantics x Language | 0.001 (0.001) | -0.005; 0.008 | 0.40 |  |
| Language x Syntax | 0.002 (0.003) | -0.003; 0.006 | 0.74 |  |
| Semantics x Scene | 0.007 (0.003) | -0.014; 0.027 | 0.61 |  |
| Scene x Syntax | 0.016 (0.007) | -0.010; 0.042 | 1.19 |  |
| Language x Scene | -0.004 (-0.003) | -0.020; 0.011 | -0.55 |  |
| Semantics x Phase | 0.003 (0.004) | -0.003; 0.009 | 1.07 |  |
| Syntax x Phase | 0.001 (0.002) | -0.003; 0.005 | 0.44 |  |
| Language x Phase | 0.001 (0.004) | -0.001; 0.003 | 1.06 |  |
| Scene x Phase | 0.003 (0.002) | -0.010; 0.016 | 0.43 |  |
| Semantics x Language x Syntax | 0.004 (0.002) | -0.013; 0.020 | 0.46 |  |
| Semantics x Scene x Syntax | -0.014 (-0.003) | -0.073; 0.045 | -0.46 |  |
| Semantics x Language x Scene | 0.013 (0.004) | -0.020; 0.045 | 0.76 |  |
| Language x Scene x Syntax | 0.010 (0.003) | -0.027; 0.047 | 0.53 |  |
| Semantics x Syntax x Phase | -0.005 (-0.002) | -0.023; 0.014 | -0.50 |  |
| Semantics x Language x Phase | -0.003 (-0.002) | -0.012; 0.006 | -0.58 |  |
| Language x Syntax x Phase | -0.002 (-0.003) | -0.008; 0.004 | -0.74 |  |
| Semantics x Scene x Phase | 0.001 (0.000) | -0.029; 0.031 | 0.08 |  |
| Scene x Syntax x Phase | -0.010 (-0.003) | -0.048; 0.028 | -0.52 |  |
| Language x Scene x Phase | 0.003 (0.001) | -0.019; 0.025 | 0.26 |  |
| Semantics x Language x Scene x Syntax | -0.039 (-0.006) | -0.116; 0.039 | -0.98 |  |
| Semantics x Language x Syntax x Phase | -0.002 (-0.001) | -0.026; 0.022 | -0.16 |  |
| Semantics x Scene x Syntax x Phase | -0.005 (-0.001) | -0.091; 0.081 | -0.12 |  |
| Semantics x Language x Scene x Phase | -0.006 (-0.001) | -0.053; 0.040 | -0.27 |  |
| Language x Scene x Syntax x Phase | -0.002 (-0.000) | -0.056; 0.053 | -0.07 |  |
| Semantics x Language x Scene x Syntax x Phase | 0.017 (0.002) | -0.096; 0.130 | 0.30 |  |

**Supplemental Material S2**

*Syntactic similarity influences scan-pattern similarity within the same language and during utterance planning, even when linearly represented as part-of-speech.*

In the main manuscript, we represented syntactic information as dependency graphs, which capture the hierarchical structure of sentences, and computed similarity using Tree Kernels. To ensure that the role of syntax was transient and restricted and our findings related to it robust to the specific method of calculation, we repeated the analysis using a surface-level syntactic measure. Here, syntactic similarity was calculated by converting each sentence into a sequence of Part-of-Speech (PoS) tags (e.g., "DET NOUN VERB") and computing the Longest Common Subsequence (LCS) between them. Unlike tree kernels, this method is sensitive to linear order but agnostic to hierarchical depth. The results of this control model are visualised in Figure S1 and reported in Table S2, largely replicating the core pattern of effects reported in our main analysis. Crucially, semantic similarity remains the dominant driver of cross-modal coordination throughout the speech generation process. Its influence is robust across visual contexts and becomes significantly stronger during the articulation phase, confirming its primacy in guiding eye movements within and across language. In contrast, the influence of syntactic similarity is language-dependent, operating primarily when sentences originate from the same language. Most importantly, the influence of syntax is transient and confined to the early planning phase as syntactic guidance observed before speaking significantly diminishes or vanishes once articulation begins. It is worth noting that while the main Tree Kernel analysis found syntactic effects to be strongest within the same visual scene context, this specific constraint did not replicate with surface-level POS sequences. This likely reflects the coarser nature of POS tags, which capture generic grammatical templates that recur frequently across different scenes, unlike dependency trees, which encode structural relations more specific to the visual event structure. Nonetheless, the primary theoretical conclusion remains robust: whether defined by deep hierarchical relationships or linear grammatical categories, syntax acts as a secondary, transient constraint during the linearization of the message.

Table S2: Linear mixed-effects model for predicting the similarity of two scan patterns (fixations mapped over a regularly spaced grid) as a function of Semantics (semantic similarity between the sentences associated with the scan patterns; dot-product over sentence embeddings), Syntax (longest common subsequence similarity between sequences of part-of-speech tags), Language (sentences use the same or a different language; different as reference Level), Scene (sentences describe the same or a different visual scene; different as reference level) and Phase (the temporal segments in the production task, distinguishing between before and during speaking; before as reference level). The participant pair (N = 2,775), item pair (N = 4,656), and word cue pair (N = 425) were the random variables introduced as intercepts.

| Predictor | $\beta$ (Std. $\beta$) | CI (2.5 %; 97.5 %) | t-value |  |
| --- | --- | --- | --- | --- |
| (Intercept) | 0.189 (1.589) | 0.185; 0.194 | 84.41*** |  |
| Semantics | 0.003 (0.003) | -0.004; 0.009 | 0.76 |  |
| Syntax | -0.014 (-0.017) | -0.017; -0.011 | -8.82*** |  |
| Language | 0.023 (0.089) | 0.019; 0.026 | 13.10*** |  |
| Scene | 0.113 (0.133) | 0.099; 0.127 | 15.71*** |  |
| Phase | -0.054 (-0.226) | -0.056; -0.052 | -55.45*** |  |
| Semantics x Syntax | 0.036 (0.025) | 0.023; 0.050 | 5.23*** |  |
| Semantics x Language | -0.056 (-0.060) | -0.066; -0.046 | -10.79*** |  |
| Language x Syntax | 0.023 (0.055) | 0.019; 0.028 | 9.84*** |  |
| Semantics x Scene | 0.004 (0.002) | -0.024; 0.033 | 0.31 |  |
| Scene x Syntax | -0.014 (-0.009) | -0.040; 0.011 | -1.10 |  |
| Language x Scene | 0.032 (0.022) | 0.012; 0.052 | 3.10** |  |
| Semantics x Phase | 0.085 (0.095) | 0.075; 0.094 | 18.15*** |  |
| Syntax x Phase | 0.067 (0.147) | 0.063; 0.071 | 30.34*** |  |
| Language x Phase | -0.009 (-0.027) | -0.012; -0.005 | -5.25*** |  |
| Scene x Phase | -0.073 (-0.060) | -0.090; -0.056 | -8.40*** |  |
| Semantics x Language x Syntax | 0.052 (0.035) | 0.033; 0.071 | 5.47*** |  |
| Semantics x Scene x Syntax | 0.115 (0.034) | 0.055; 0.174 | 3.80*** |  |
| Semantics x Language x Scene | 0.028 (0.009) | -0.018; 0.075 | 1.19 |  |
| Language x Scene x Syntax | -0.041 (-0.016) | -0.079; -0.003 | -2.10* |  |
| Semantics x Syntax x Phase | -0.134 (-0.084) | -0.154; -0.115 | -13.55*** |  |
| Semantics x Language x Phase | 0.129 (0.104) | 0.114; 0.143 | 17.55*** |  |
| Language x Syntax x Phase | -0.062 (-0.112) | -0.068; -0.055 | -19.36*** |  |
| Semantics x Scene x Phase | -0.002 (-0.001) | -0.043; 0.039 | -0.08 |  |
| Scene x Syntax x Phase | 0.050 (0.021) | 0.013; 0.088 | 2.63** |  |
| Language x Scene x Phase | -0.027 (-0.013) | -0.057; 0.003 | -1.79 |  |
| Semantics x Language x Scene x Syntax | -0.050 (-0.011) | -0.133; 0.032 | -1.20 |  |
| Semantics x Language x Syntax x Phase | -0.090 (-0.045) | -0.116; -0.063 | -6.59*** |  |
| Semantics x Scene x Syntax x Phase | -0.046 (-0.009) | -0.132; 0.040 | -1.04 |  |
| Semantics x Language x Scene x Phase | -0.081 (-0.018) | -0.149; -0.013 | -2.35* |  |
| Language x Scene x Syntax x Phase | 0.025 (0.007) | -0.030; 0.081 | 0.89 |  |
| Semantics x Language x Scene x Syntax x Phase | 0.101 (0.015) | -0.020; 0.221 | 1.64 |  |

*Figure S1: Scan pattern similarity as a function of syntactic similarity (with longest common sequence on part-of-speech tags as similarity measure). Within each panel, we compute the mean of scan pattern similarity (y-axis) over regularly spaced bins of syntactic similarity (x-axis, ranging from 0 to 1 in increments of 0.1) and indicate the trend of the mean (as a loess line). Line colour, font, and symbol type indicate whether the similarities have been computed within the same scene (blue triangle), between different scenes (red circle), or in the shuffled condition (grey square). All comparisons between the different languages are displayed across the panels (English; Portuguese; Japanese; English--Portuguese; English--Japanese; Portuguese--Japanese), while Phases are compared as rows, with top panels showing similarities computed before speaking, while the bottom panels show similarities during speaking.*

*
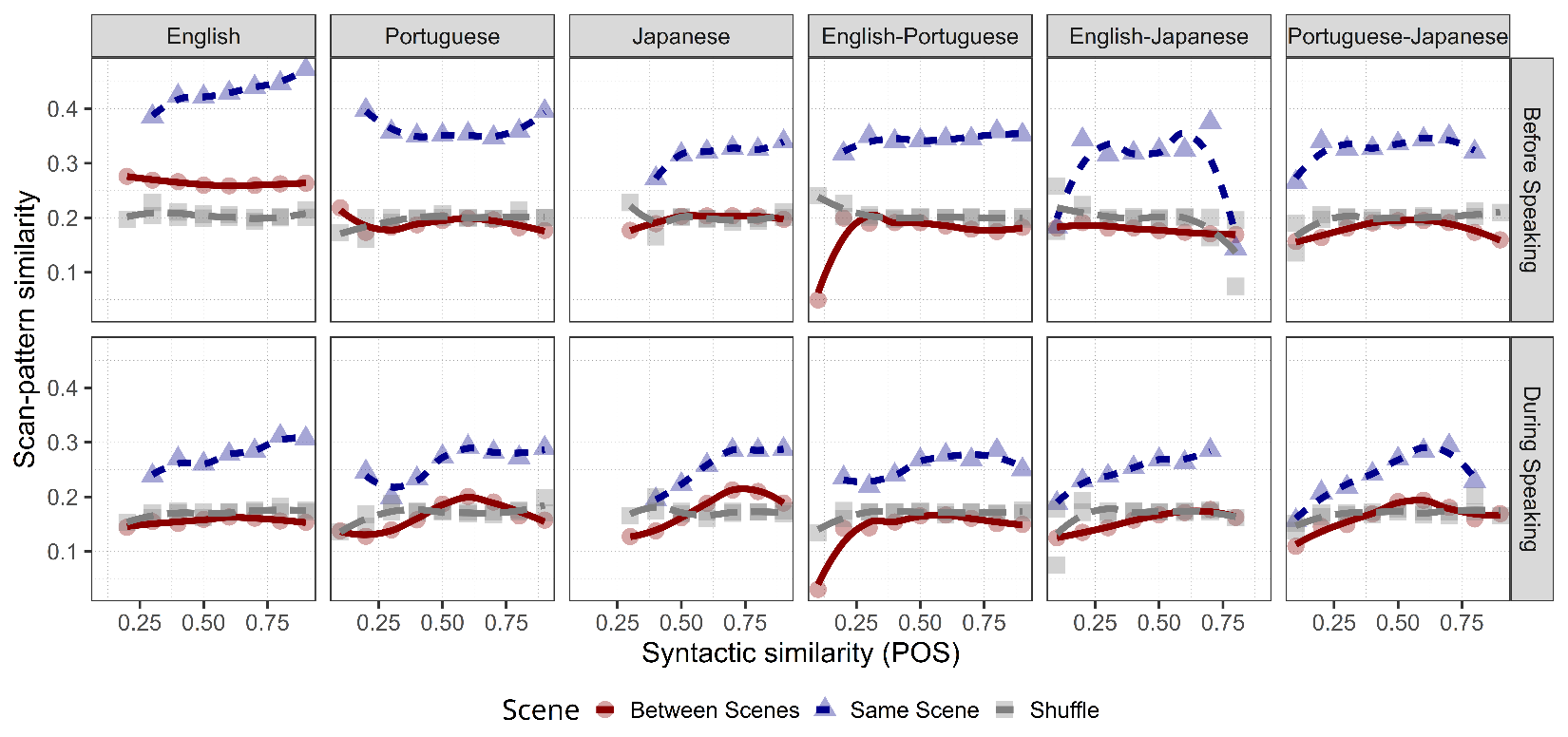
*
